# REFOCUS-PULSAR Recovery-Oriented Practice Training in Adult Primary Mental Health Care: Exploratory Findings Including From a Pretest–Posttest Evaluation

**DOI:** 10.3389/fpsyt.2021.625408

**Published:** 2021-03-11

**Authors:** Joanne C. Enticott, Frances Shawyer, Lisa Mary Brophy, Grant Russell, Danielle Mazza, Elisabeth Wilson-Evered, Penelope June Weller, Mike Slade, Vrinda Edan, Graham Nicholas Meadows

**Affiliations:** ^1^Southern Synergy, Department of Psychiatry, Monash University, Melbourne, VIC, Australia; ^2^Monash Centre for Health Research and Implementation, Monash University, Clayton, VIC, Australia; ^3^School of Allied Health, Human Services and Sport, College of Science, Health and Engineering, La Trobe University, Melbourne, VIC, Australia; ^4^Centre for Mental Health, School of Population and Global Health, University of Melbourne, Melbourne, VIC, Australia; ^5^Department of General Practice, Monash University, Melbourne, VIC, Australia; ^6^Institute of Health and Sport, Victoria University, Melbourne, VIC, Australia; ^7^Graduate School of Business and Law, College of Business, RMIT University, Melbourne, VIC, Australia; ^8^School of Health Sciences, University of Nottingham, Nottingham, United Kingdom; ^9^Faculty of Medicine, Dentistry and Health Sciences, University of Melbourne, VIC, Australia; ^10^Monash Health, Clayton, VIC, Australia

**Keywords:** primary care (MeSH), implementation, general practice (MeSH), mental health, recovery-oriented practice

## Abstract

**Objectives:** Australian general practitioners (GPs) are pivotal in mental health care. The REFOCUS-PULSAR (Principles Unite Local Services Assisting Recovery) primary care study aimed to improve personal recovery outcomes in adults with mental health problems consulting GPs.

**Design:** Modified from an intended stepped-wedge cluster study, an exploratory (pre- and post-intervention) design employed cross-sectional surveys of patients consulting GPs.

**Setting:** Eighteen primary care sites (clusters) in Victoria, Australia in 2013–2017.

**Participants:** From 30 GPs recruited, 23 participated (76%), with 235 patient surveys returned from adults aged <75 years receiving mental health care.

**Intervention:** A co-delivered face-to-face training intervention for GPs in recovery-oriented practice (ROP), with personal recovery a key focus, used multimedia, mnemonics, and targeted interview schedules to encourage ROP—with availability of support sessions for 1 year.

**Outcome Measures:** Primary: the Questionnaire about the Process of Recovery full-scale score (outcome). Secondary: INSPIRE (experience), Warwick-Edinburgh Mental Well-being Scale (WEMWBS) and Kessler Psychological Distress Scale (K10) (outcomes). Other: General-practice-Users Perceived-need Inventory (experience).

**Results:** Small positive significant effects indicated primary-outcome post-intervention improvements [*t*-test (233) = −2.23, *p* = 0.01], also improvement in two secondary outcomes (WEMWBS *t*(233) = −2.12, *p* = 0.02 and K10 *t*(233) = 2.44, *p* = 0.01). More patients post-intervention reported “no need” for further help from their GP; but in those reporting needs, there was greater unmet need for counseling.

**Conclusions:** ROP implementation, internationally influential in specialist mental health care, here is explored in primary care where it has had less attention. These exploratory findings suggest better patient outcomes followed introducing GPs to ROP in routine practice conditions. Higher unmet need for counseling post-intervention reported by patients might be a sign of limited supply despite ROP facilitating better identification of needs. Challenges in project implementation means that these findings carry risks of bias and flag the importance establishing research infrastructure in primary care.

**Clinical Trial Registration:** www.clinicaltrials.gov/, The Australian and New Zealand Clinical Trial Registry Identifier: ACTRN12614001312639.

## Introduction

Mental health-related problems have latterly been reported as the most common reason patients consult general practitioners (GPs) in Australia, increasing from 10.8% of all consultations in 2007–2008 to 12.4% in 2015–2016 (1). This trend is unsurprising given that mental disorders yearly affect 1-in-5 Australians (2, 3). Australian government legislation has supported GPs' central role in the management of mental health problems through the Medicare Better Access to Mental Healthcare initiative (“Better Access”). This initiative, implemented nationally in late 2006, subsidizes access to eligible GPs, psychiatrists, psychologists and other mental health services (4). GPs prescribe antidepressant medications at a rate of 61.6 per 100 mental health consultations and make referrals for 18.8 per 100, with more than half of referrals made to psychologists in 2015–2016 (1). Meadows et al. (5) examined data from all Australia-wide Better Access services and showed that GP mental health services were far more equitably delivered than services delivered by psychiatrists or clinical psychologists, highlighting the important, frontline role that GPs play in the care of all Australians for mental health conditions (5).

Recovery-oriented practice (ROP) is an approach to mental health care that has gained influence over recent times. The approach involves facilitating a process of change through which individuals are supported to build and live fulfilling and meaningful lives, with or without the continuing presence of mental health issues (6, 7). The meaning of the term “recovery” in this context is distinguished from clinical recovery and has been summarized as:

*A deeply personal, unique process of changing one's attitudes, values, feelings, goals, skills and roles. It is a way of living a satisfying, hopeful and contributing life even with limitations caused by the illness. Recovery involves the development of new meaning and purpose in one's life as one grows beyond the catastrophic effects of mental illness* (8).

ROP is a patient-centered, strength-based approach to supporting people living with a diagnosis of mental illness. State, Territory and national policies (9, 10) in Australia feature a recovery focus, which is also embraced by countries including Canada, England, Ireland, Germany, The Netherlands, New Zealand, Norway, Scotland, and The United States where ROP is being applied to mental health service delivery in diverse settings (11–14).

ROP studies typically have been conducted in contexts involving various forms of specialist mental health care in community settings, whether delivered through publicly funded services or non-government organizations (NGOs). A recent trial of a training intervention for ROP advanced the field by showing improved personal recovery outcomes for consumers of specialist mental health services exposed—across both these sectors—to the training intervention (15). We found only one study in the literature regarding primary care and ROP and this was not an intervention study. This survey of 577 primary care patients with depressive symptoms sought to identify the patient perceived importance and benefits of having access to a written plan to recover from “depression, stress, or worries.” The majority of respondents thought that having access to written plans for recovery was very important. Many of the benefits they reported, such as hope, direction, and independence were consistent with a recovery orientation and the authors concluded that written plans for recovery could support recovery-oriented primary care for depression (16).

To our knowledge, no study has been published that has examined whether interventions promoting recovery-oriented practice in general practice settings can improve outcomes for patients. Given the current clear expectation in policy throughout Australia that recovery be a core part of mental health care practice, and that the GP is the service provider most commonly consulted for mental health problems, this situation represents a critical area for intervention (9, 17).

The REFOCUS-PULSAR (Principles Unite Local Services Assisting Recovery) Primary Care trial involved a training intervention for GPs in recovery-oriented practice (ROP) in Victoria, Australia. Conducted in parallel with a similarly named project in secondary public mental health and non-government community services (15), the aim of the primary care intervention was to influence GP care provision so as to improve personal recovery in patients consulting study GPs for mental health issues. The primary research question of the study was whether patients of GPs sampled after these GPs have received receiving training in ROP report greater personal recovery as self-assessed using a Patient Reported Outcome Measure (PROM). The secondary research PROM question was whether in the same intervention context, do these patients report improvements on health and well-being PROMs? We also explored as Patient Reported Experience Measures (PREMs) whether patients of GPs receiving training in ROP reported improvements on measures of recovery support and perceived need for care.

## Methods

### Study Design

#### Original Design (Planned)

The design was originally planned as a stepped-wedge cluster randomized controlled trial (cRCT) of training in ROP delivered to GPs, using cross-sectional surveys to collect data from patients of participating GPs at three time points: baseline, and again at the end of Steps 1 and 2. Primary care sites (clusters) were randomly allocated to one of two steps (9 months apart) to start the GP training (18). For clusters allocated to step 1, data collection involved one pre-training data collection point and two post-training data collection points. For clusters allocated to step 2, data collection involved two pre-training data collection points and one post-training data collection point.

#### Adapted Design (Used)

**Figure 1:**
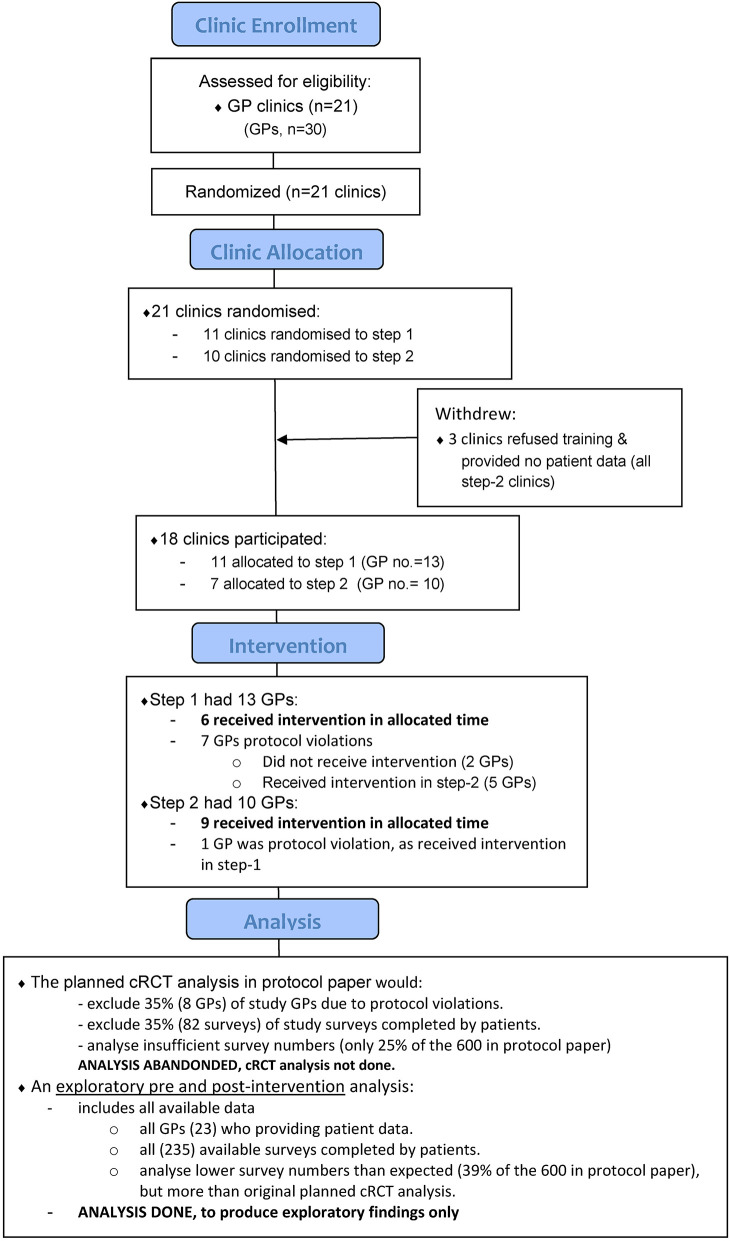
Participating clinic and general practitioner (GP) flow diagram. GPs allocated to step 1 were planned to undergo training first, and then approximately 9 months later, the step 2 GPs trained. However, protocol violations occurred and sometimes GPs in either group trained together. Together with Supplementary File 1, it shows the original stepped-wedge cRCT intervention design was not achieved. Instead, exploratory pre and post-intervention analyses were done.

Implementation of the intended design was not achieved as a result of difficulties with GP recruitment, patient data collection and intervention protocol adherence (see section “Results”). Data collection proceeded over three time points but the study steps were reduced from the 12 months originally planned to 9 months and data collection occurred over 6 month periods, 3 months apart [see (18), Figure 1]. To enable data analysis in the context of low response rates, the design was adjusted after the completion of participant recruitment as a pre- and post- intervention design by collapsing data from the three data collection points. In line with the original design (18) and with similar argument for this as set out in the specialist study design (15, 19), the study employed cross-sectional data from different, anonymous, patient samples. We acknowledge this as an intrinsically inferior design to that planned and so frame the findings as exploratory (20).

The decision to amend the analysis design was made following the recommendation by the Research Module Committee at the end of data collection, and was confirmed by the Steering Committee and Principal investigator (GM). All adaptations to the study protocol were approved by the appropriate Module Committee governing the relevant aspect of the project [see “Study Leadership” section in protocol (19)], received ethics' committee approval (18), were communicated to and endorsed by the funding body.

A qualitative and process evaluation were embedded within the GP trial (18) and results will be reported separately.

### Patient and Public Involvement

We refer to people who experience mental illness and engage with services as “consumers” (15, 19) but in the Primary Care project described in this paper, the term “patient” will be used as more representative of regular usage in this context. The broader PULSAR research program was enhanced by consumer involvement from the outset (VE) and an overarching principle that all consumer data collection was overseen by consumer researchers. Study components were co-designed with a consumer academic (VE) and the involvement of the PULSAR Lived Experience Advisory Panel [LEAP; see Leadership structure (19)]. The GP training was delivered by mental health clinicians, including experienced trainers from the study team, with co-delivery by consumer trainers. Consumer trainers were people with lived experience of their own recovery journey and experience working in support and advocacy roles.

### Clusters

In the original design, clusters (18) were participating general practices and community health centers that employ GPs. Each cluster could include more than one GP. Control clusters were those that were yet to receive the intervention. There were no study restrictions on the care provided in control phases.

### Randomization

Random allocation was used to nominate each cluster to one of two steps (9 months apart) to complete their GPs training (18). Breaking the cluster intervention code necessarily occurred after randomization so that training dates could be organized at clusters. Only key people involved with the organization and delivery of the training were informed of the intervention/training schedule.

### Study Setting and Recruitment

#### GP Recruitment

Eighteen primary care sites (clusters) were recruited. The study catchment was expanded from the original plan to reach the target for GP recruitment, which was 30 GPs, as described in the protocol paper (18). The final area of recruitment consisted of approximately 1,392 million Victorians, or 24% of Victoria's population including affluent areas, semi-rural growth corridors and regions with disproportionately high numbers of retirees and older Australians. Included was one of the most socially and financially disadvantaged areas in Australia, which ranks within the lowest quintile on the Index of Relative Socioeconomic Disadvantage (21), a composite measure of relative area-based socioeconomic status where lower quintile rankings indicate greater disadvantage.

#### GP Eligibility Criteria

To be eligible, practice sites were required to be accredited by the National Australian General Practice Accreditation scheme (22). GP eligibility criteria were: having worked at their current practice for at least 12 months, with minimum 2.5 days per week at the study site and a majority of that work in generalist primary care. Enrolment involved committing to participate in the ROP intervention, identifying eligible patients, and distributing study invitation letters and surveys to potential patient participants. Each primary care site received a base remuneration of $500 for committing resources to help offset practice administration costs involved in recruitment and an additional $25 for the successful recruitment of each eligible patient.

#### Patient Recruitment

Participating GPs were responsible for organizing patient recruitment at their site. We intended that each GP would recruit a minimum 30 patients during the two study steps, each of which proceeded for 9 months. For more detail, see the study protocol publication (18). Study GPs were required to identify eligible patients supported by instructions provided by researchers in direct personal briefings and a handout summarizing the patient eligibility criteria described below. The participating GP at each cluster site coordinated the identification of eligible patients from the service billing and clinical software then oversaw a mailout of an invitation letter, participant information and consent form and survey to eligible patients. This method was adopted to ensure recruitment was independent from the researchers, to maintain privacy of participants. GPs were provided with practical assistance to limit burden, such as patient information flyers and posters and template recruitment letters and scripts. The surveys asked patients for demographic details and completion of the measures listed below. Surveys were provided to patients with a return, stamped envelope to enable the return of completed surveys to the researchers. GP-led recruitment was supplemented by other recruitment strategies flexibly employed to promote patient response, depending on the needs of each practice. These included having additional survey packs available at sites, including flyers and posters in the practice waiting room inviting patients of participating GPs to join the study, GPs or practice staff handing information flyers or the survey packs directly to eligible patients.

#### Patient Eligibility Criteria

Base inclusion criteria for patient participants were: aged 18 years and over; aged <75 years of age; proficient in English; able to provide informed consent; patients of a participating GP, that is, consult with the GP in at least 50% of their visits to the practice or is identified by the GP as a patient. In addition to the base criteria, GPs were advised to recruit 10 patients at each of the three time periods, and seven were to have either: (a) a recent mental health plan or a review of a mental health plan under the Medicare Better Access initiative in the last 3 months, or, (b) to have been prescribed any class of antidepressant medication on a continuing basis as treatment for a mental illness in the previous 3 months. Three patients were required to have: (a) a diagnosis of psychosis (e.g., schizophrenia, schizoaffective disorder, bipolar disorder), or (b) had been prescribed antipsychotic medication in the previous 6 months.

### Ethics

This study was approved by the Human Research Ethics Committees of Monash Health (approval number 14146B) and Monash University (project approval number CF14/2422−2014001304 and CF15/266−2015000120).

### Intervention

The training in ROP was designed to encourage living beyond the challenges of mental illness by promoting connectedness, hope, identity, meaning, and empowerment through clinician-patient working practices such as understanding patient values and treatment preferences, assessing strengths and supporting goal striving (23, 24). The intervention was designed to meet criteria for Mental Health Skills Training (MHST), so qualifying the participants for specific increased service rebates under the Australian Medicare health insurance system, specifically in the Better Access to Mental Health Care funding scheme (“Better Access”) (25). In line with the modular pathway for this training, the first part of the intervention consisted of two training modules (18). Trainers in each instance included a psychiatrist and a person with lived experience of mental health problems and training experience. These two were present through the whole workshop period, with varying assignment of leadership for specific elements—throughout concentrating on a collaborative mutually supportive approach to delivery (15). An experienced family/carer worker also participated, providing a minimum of 1 h of content over the total of 8 h of training as required for MHST. The intervention drew heavily on the REFOCUS program (26), the General Practitioner Mental Health Treatment Plan-Recovery (available from authors upon request) and CLIPP program (27).

Standardized training packs (including videos) with checklists supported the provision of standardized content as grouped below. Trainers were asked to keep a record of training and to record any deviations from the schedule and use of materials. Most participants were GPs, also some practices were trained at their specific sites in which case the inclusion was broadened to include associated psychologists, nurses and practice managers. A training pack to take away included a desk-reference manual for the REFOCUS-PULSAR approach in primary care (28).

#### Modular MHST Component

1. Core Module (3.5 h). Required and quite closely stipulated for MHST, learning objectives including increased skill in recognizing and assessing common mental illnesses, a greater working knowledge of the Better Access initiative, mental health treatment planning and increased knowledge of local mental healthcare services and resources available to GPs. This intervention placed core training in a recovery-oriented framework with a focus on: operationalizing ROP in general practice; and enhanced understanding of the perspective of consumers and carers in the provision of mental healthcare. Some GPs who already had MHST qualifications did not take part in this activity and went straight to the second module.

2. Clinical Enhancement Module (CEM; 4 h). This component provided the opportunity to apply knowledge gained in the Core Module within the specific context of Schizophrenia. Learning objectives of the CEM include: development of skills in the detection and assessment of Schizophrenia; an ability to apply the principles of ROP to treatment planning and monitoring; the ability to develop recovery-focused mental health treatment plans; and an applied understanding of review processes and relapse prevention strategies for mental illness within a ROP framework. Mental Health Skills Training criteria required for access to subsidized mental health items under Medicare are met by participation in both modules, along with preparatory and reinforcing exercises.

#### Optional Active Learning Sessions

3. PALS (optional). REFOCUS-PULSAR active learning sessions. GPs and other professionals who received the training were invited to participate in monthly 1-h online sessions called “PALS (PULSAR Active Learning Sessions)” with a consultant specialist psychiatrist to review, reflect and share their experiences in the implementation of ROP. These sessions provided an interactive learning environment for supporting practice-based implementation of learning from the REFOCUS-PULSAR resources and training package.

### Outcome Measures

The primary outcome, positive progress in personal recovery, here classed as a PROM, was identified using the Questionnaire about the Process of Recovery (QPR); a 22 item self-report instrument (29).

Secondary PROMS were sourced from: the Warwick-Edinburgh Mental Well-being Scale (WEMWBS), which assesses subjective well-being and psychological functioning (30); and the Kessler Psychological Distress Scale (K10), which assesses mental health (31). Among PREMs, the INSPIRE questionnaire seeks responses from patients on how their GP supports their recovery in (1) recovery domains the patient regards as important; and (2) overall relationship terms. The General-practice-Users Perceived-need Inventory (GUPI), assessed the patients' estimate of mental health care needs and meeting those needs across five types of help (Information, Medication, Counseling, Practical Issues, and Skills training). A final barriers question asked participants for the reasons that may have stopped them in the last few weeks from receiving any of the kind of help listed or from having as much help as needed. Here, more than one option can be selected by each participant.

Health economic and service utilization data will be reported in a separate health economic evaluation and are not included in this paper.

### Implementation Issues and Statistical Analyses

The original design was a stepped-wedge cRCT, whereby clusters were randomly allocated a start date for training in either step 1 or step 2. The cluster randomization was done, and research staff attempted to organize and conduct training with each GP within their allocated period. However, as seen in Figure 1 and Supplementary File 1, the two separate training periods did not eventuate and GPs in either group were trained together. Challenges were encountered when arranging GPs to attend within their allocated training period because re-scheduling was requested by some GPs, occasionally multiple times. Additionally, some GPs confirmed a training date, and then did not attend. Also, while there were instances of highly effective recruitment in some settings, overall, the design eventuated to involve an over-optimism about recruitment capacities. Base remuneration was increased through the course of the project (from $200 to $500) (18), however, patient recruitment in participating practices fell considerably below the hoped-for target.

Descriptive statistics summarize the key demographic factors of patients who completed study surveys. Due to numbers of patient surveys received, and to preserve anonymity, patient results are not summarized at the practice level. Descriptive statistics summarize the characteristics used in the stratified randomization of the clusters. The distribution of all QPR scores is reported to compare with another recent large sample (15).

For purpose of protocol compliance, we report intention-to-treat numbers of patient surveys using the original cRCT timeframes. Survey responses received, however, were insufficient to allow analysis within this design, so a pre-post (PP) analysis was conducted. The PP analyses utilize all available data making the best use of the smaller than expected data set. Our exploratory analyses include *t*-tests on the cross-sectional data collected, and the results of which are not inferential but rather exploratory and hypothesis generating (20, 32).

As sensitivity analyses, the PP primary outcome data was analyzed using propensity scores estimated by the conditional treatment probability using maximum likelihood (probit regression), with independent factors of GP clinic, gender, age, country of birth (Australia: Yes/No). Propensity score and Mahalanobis matching drawn with replacement were then applied using the stata code PSmatch2. Four matching strategies were investigated: nearest neighbor 1-to-1; nearest neighbor 1-to-2; Mahalanobis 1-to-1, and; Mahalanobis 1-to-2. These matching strategies however excluded 10–13% of the available data (i.e., in the available primary outcomes from 235 subjects, the four matched analyses included data from: 206, 206, 210, and 208 individuals, respectively). Incomplete matching particularly when done with relatively small data sets such as in this paper, has been reported to contribute toward biased treatment estimates (33). Therefore, the pre- and post-design was chosen as the main analysis as it used all the available data, and the propensity scores analyses included as sensitivity analyses.

## Results

### GP Recruitment and Participation

We recruited 30 GPs from 21 practices; however, seven GPs (3 sites) withdrew without receiving the intervention or contributing any data and were therefore excluded from the analysis. Twenty-one of 23 participating GPs were trained in 13 months between March 2015 and April 2016. Participating GPs were located over 18 practices; one practice had three GPs involved, three practices had two GPs, and all remaining practices had one GP.

In the 18 clusters, there were 17 privately owned general practices and 1 community health center; practice sizes by equivalent full time (EFT) were: >5 EFT employed GPs: 12 practices; 2–5 EFT employed GPs: 4 practices; <2 EFT employed GPs: 2 practices and 5 practices with a specialist focus clinic, such as youth sexual health and HIV/AIDS.

### Patient Surveys

We received 235 surveys from patients of the 23 GPs from March 2015 to May 2017, see Table 1 (cf., planned minimum of 600 patient surveys (30 per GP) (18)). Thirty-three percent (6/18) of practices contributed 1–3 patient surveys each. Only one GP exceeded the 30 minimum target per GP with 51 patient surveys. From the remaining 22 GPs, we received between 1 and 24 patient surveys per GP, with a median of 9.

**Table 1:** Patient survey numbers and demographics.

**Variable**		**Pre** **(*n* = 136)**	**Post** **(*n* = 99)**	**Total** **(*n* = 235)**
		**Years**	**Years**	**Years**
**Age**	Mean	41.6	44.4	42.8
	Median	42.5	45.0	44.0
	Standard deviation	13.8	14.0	13.9
	Range (min-max)	18–75	21–75	18–75
		***n*** **(%)**	***n*** **(%)**	***n*** **(%)**
**Gender**	Female	88 (64.7)	62 (62.6)	150 (63.8)
	Male	48 (35.3)	36 (36.4)	84 (35.7)
	*Not listed*	*0 (0)*	*1 (1.0)*	*1 (1.0)*
**Country of birth**	Australia	111 (81.6)	79 (79.8)	190 (80.9)
	*Other*	*25 (28.4)*	*20 (20.2)*	*45 (19.1)*
**Main language spoken at**	English	118 (86.8)	91 (91.9)	209 (88.9)
**Home**	Other	11 (8.1)	2 (2.0)	13 (5.5)
	*Not listed*	*7 (5.1)*	*6 (6.1)*	*13 (5.5)*
**Australian indigenous**	Yes	8 (5.9)	4 (4.0)	12 (5.1)
**Marital status**	Single	57 (41.9)	49 (49.5)	106 (45.1)
	Married/De Facto	49 (36.0)	34 (34.4)	83 (35.3)
	Separated/Divorced	25 (18.4)	14 (14.1)	39 (16.6)
	Widowed	2 (1.5)	1 (1.0)	3 (1.3)
	*Not listed*	*3 (2.2)*	*1 (1.0)*	*34 (1.7)*
**Living arrangement**	Living with parents	26 (19.1)	13 (13.1)	39 (16.6)
(multiple responses allowed)	Living with siblings	10 (7.4)	3 (3.0)	12 (5.5)
	Living with a partner	49 (36.0)	35 (35.4)	84 (35.7)
	Living with children	30 (22.1)	20 (20.2)	50 (21.3)
	Living with friends	4 (2.9)	5 (5.1)	9 (3.8)
	Living in shared accommodation	5 (3.7)	13 (13.1)	18 (7.7)
	Living in crisis accommodation	3 (2.2)	0	3 (1.3)
	Living in support housing	8 (5.9)	3 (3.0)	11 (4.7)
	Homeless	1 (0.7)	0	1 (0.4)
	Living alone	30 (22.1)	32 (32.3)	62 (26.4)
	*Other*	*8 (5.9)*	*3 (3.0)*	*11 (4.7)*
**Highest education level**	Primary school	2 (1.5)	3 (3.0)	5 (2.1)
	Secondary school (junior)	29 (21.3)	22 (22.2)	51 (21.7)
	Secondary school (senior)	33 (24.3)	26 (26.3)	59 (25.1)
	Post-secondary school	71 (52.2)	51 (51.5)	122 (51.9)
	*Not listed*	*2 (1.5)*	*0 (0)*	*2 (0.9)*
**Medications for mental health**	Yes	117 (86.0)	68 (68.7)	185 (78.7)

The distribution of QPR scores from the 235 patients is in Supplementary File 2. QPR mean was 54.7, standard deviation of 15.2. Results compare with another sample of 942 patients using secondary mental health care services (25), which had QPR mean of 54.0 and standard deviation of 16.2. A two-sample Kolmogorov–Smirnov test for equality of distribution showed these QPR datasets appear to have the same distribution function (D-statistic= 0.047, *p* < 0.80).

See Table 2 for the number of patient surveys completed within the intention-to-treat timeframes and two types of protocol violations including surveys completed outside the specified ITT periods, and surveys received from practices not matching their planned ITT intervention status. Supplementary File 1 shows the number of GPs not trained within their allocated step period, hence leading to protocol violations.

**Table 2:** Breakdown of the 235 patient surveys in each intention-to-treat time-point.

**ITT periods**		**Early (step 1)**	**Late(step 2)**	**Total**
**T0**	November 15, 2014–May 15, 2015	21	8	29
**T1**	August 15, 2015–February 15, 2016	5 [15]^pv1^	32	52
**T2**	May 15, 2016–November 15, 2016	36 [1] ^pv1^	31 [4] ^pv1^	72
**ITT-Total**		**78**	**75**	**153**
***Outside ITT periods***		[44] ^pv2^	[38] ^pv2^	[82]
***KEY:*** ***ITT intervention status***		***Non**-intervention periods shaded light-gray*		
		*P**ost**-intervention periods shaded darker-gray*		

*ITT protocol violations shown in square brackets. Excludes one survey returned with no measures completed. Two types of protocol violations are shown (PV1) surveys completed outside the specified ITT periods, and (PV2) surveys received from practices not matching their planned ITT intervention status. For example, at time T1, the ITT schedule required step 1 practices to be post-intervention; however, there were 15 surveys received from practices not yet in receipt of the intervention*.

### Pre and Post-intervention (PP) Analyses

*t*-Tests showed significant improvement post-intervention in the primary outcome (*t*(233) = −2.23, *p* = 0.01) and two of three secondary outcomes (WEMWBS *t*(233) = −2.12, *p* = 0.02 and K10 *t*(233) = 2.44, *p* = 0.01). Small effect sizes, with Cohen's d ranging between 0.29 and 0.32, were observed for all outcomes except the INSPIRE (see Table 3). The four sensitivity analyses for the primary outcome using propensity score matching are in Supplementary File 3, and show all matched analyses produced slightly larger intervention effects than the main analysis, and all were significant at *p* < 0.05.

**Table 3:** Pre and post (PP) intervention outcome differences.

			**Pre-intervention cross-sectional sample**		**Post-intervention cross-sectional sample**		**Pre minus Post**	***t*-Test**	**Effect size**
			**Total *N***	**Mean (SD)**	**Total *N***	**Mean (SD)**	**Mean difference**	***t*-Statistic and *p*-value**	**Cohen's *d***
**Primary outcome**	**QPR**		136	52.8 (15.6)	99	57.3 (14.3)	−4.4	−2.23, *p* = 0.01^*^	0.29
**Secondary outcomes**	**INSPIRE**	**INSPIRE-S**	135	68.8 (22.2)	92	65.9 (24.6)	2.9	0.86, *p* = 0.39	
		**INSPIRE-R**	135	81.9 (18.4)	95	82.7 (16.6)	−0.8	−0.35, *p* = 0.36	
	**WEMWBS**		136	40.0 (11.4)	99	43.1 (10.8)	−3.1	−2.12, *p* = 0.02^*^	0.28
	**K10**		136	29.3 (8.9)	99	26.6 (8.0)	2.8	2.44, *p* = 0.01^*^	0.32
		**Total** ***N***	***N*** **(%)**	**Total** ***N***	***N*** **(%)**	**% difference**	***t*****-Statistic and** ***p*****-value**	**Overall change**	
GUPI:Prevalence of needs perceived	**Unmet need**	136	120 (17.6)	99	165 (33.3)	−15.7	−2.8, *p* = 0.006^*^	*More than 15% increase*	
	**Need met**	135	384 (56.5)	99	98 (19.8)	36.7	5.6, *p* < 0.0001^*^	*Decreased by more than 36%*	
	**No need**	135	169 (24.9)	99	224 (45.3)	−20.4	−3.3, *p* = 0.001^*^	*More than 20% increase*	
GUPI:Prevalence of needs perceived as *unmet* for each variable.	**Information**	136	22 (16.2)	99	18 (18.2)	−2.0	−0.4, *p* = 0.69		
	**Medication**	135	17 (12.5)	99	19 (19.2)	−6.7	−1.4, *p* = 0.16		
	**Counseling**	135	22 (16.2)	99	27 (27.3)	−11.1	−2.1, *p* = 0.04^*^	*More than 10% increase*	
	**Practical Issues**	136	30 (12.8)	99	17 (17.2)	−4.4	−0.9, *p* = 0.35		
	**Skills training**	136	29 (12.3)	99	17 (17.2)	−4.9	1.1, *p* = 0.29		

*The QPR, WEMWBS and K10 had improved mean results after the intervention. GUPI shows an increase by 10% post-intervention in the proportion of participants who reported an unmet need for counseling*.

* *Significant difference at p < 0.05*.

GUPI results are in Tables 3, 4. There was a significant increase (10% increase post-intervention) in the proportion who reported an unmet need for counseling (*t*(233) = −2.1, *p* = 0.04), see Table 4. Whilst in the other categories (Information, Medication, Practical Issues, and Skills training) there were no significant differences between the two samples.

**Table 4:** Reasons indicated by participants that stopped them from getting any of the kinds of help listed in the GUPI or from getting as much help as needed in the last few weeks.

**GUPI**	**Pre-intervention (*****n*** **= 136)**					**Post-intervention (*****n*** **= 99)**				
**Perceived reasons**	**Information (%)**	**Medication (%)**	**Counseling (%)**	**Practical issues(%)**	**Skills training (%)**	**Information(%)**	**Medication (%)**	**Counseling(%)**	**Practical issues (%)**	**Skills training** **(%)**
Not Applicable^**^	22.1	22.1	22.1	22.1	22.1	32.3	32.3	32.3	32.3	32.3
I preferred to manage myself^*^	25.9	25.7	25.7	25.7	25.7	36.4	36.4	36.4	36.4	36.4
I didn't think anything would help	19.1	19.1	19.1	19.1	19.1	21.2	21.2	21.2	21.2	21.2
I didn't know where to get help	10.3	10.3	10.3	9.6	10.3	11.1	11.1	11.1	11.1	11.1
I was afraid to ask for help or what others would think of me	15.4	15.4	15.4	15.4	15.4	18.2	18.2	18.2	18.2	18.2
I couldn't afford the money	22.1	22.1	22.1	22.1	22.1	23.2	23.2	23.2	23.2	23.2
I asked but didn't get help	11.0	11.0	11.0	11.0	11.0	12.1	12.1	12.1	12.1	12.1

*Note, more than one option can be selected by participants*.

** *Significant increase between 22.1 and 32.3% (z-statistic = −1.75, p = 0.04)*.

* *Significant increase between 25.9 and 36.4% (z-statistic = −1.73, p = 0.04)*.

Overall, more people in the post-intervention sample (20% increase post-intervention) reported having no need for further help from their GP (*t*(232) = −3.3, *p* < 0.01). However, in those with needs for further help, there were fewer participants reporting their needs were met (36% decrease post-intervention) (*t*(232) = 5.6, *p* < 0.01); and more reporting their needs were unmet (15% increase post-intervention) (*t*(233) = −2.8, *p* < 0.01), see Table 3.

Reasons indicated by participants that stopped them from receiving any of the types of help listed in the GUPI or from having as much help as needed in the last few weeks, are shown in Table 3. The proportion who indicated this section was “not applicable” was 22% in the first sample and increased to 32% in post-intervention sample (*z*-statistic = −1.72, *p* = 0.04). The proportion who indicated the reason was “I prefer to manage myself” was around 26% in the first sample and increased to 36% in the post-intervention sample (*z*-statistic = −1.73, *p* = 0.04).

## Discussion

Better patient outcomes were suggested following the REFOCUS-PULSAR GP training intervention as the primary outcome and two of the three secondary outcomes significantly improved from pre- to post-intervention, albeit achieving only a small effect size. This finding, while acknowledged as exploratory only, is consistent with recent findings of a similar intervention delivered by the same researchers within a successfully delivered pragmatic stepped-wedge cRCT but in different professional and service contexts. The latter showed significant but small improvements in the same primary outcome measured from consumers of specialist mental health services (15). The findings reported here suggest the intervention has the potential to extend ROP to GPs throughout the community.

The GUPI assesses a patients' estimate of mental health care needs and the degree to which care meets those needs. The findings show the profiles of needs within the pre- and post-intervention samples, which were significantly different. Proportionally there appeared to be more participants in the post-intervention sample who reported having no need for further help from their GP. However, in those with needs, there was an increase in the post-intervention proportion with unmet needs, and sub-category examinations revealed this post-intervention unmet need increase was markedly for counseling. These differences suggest that REFOCUS-PULSAR may have been effective in supporting the GPs with managing patients with mental health issues by identifying a wider range of needs than might otherwise had been ascertained. However, the service volume availability that would enable those needs to be met were unchanged. This occurrence then may raise possible dilemmas for GPs, about whether to discuss (or not) with patients about any identified needs where the services are not available to meet them. For example, clinical psychologist services funded by Medicare are three-fold more times accessed in areas of high socioeconomic advantage compared to disadvantaged areas despite higher need in these areas (5, 18). A conjecture formed by these exploratory findings and consistent with emerging literature internationally (34) and which we hope to examine in future research (35) is this: *Is it actually more attractive for a GP to emphasize the potency of pharmaceutical interventions for less affluent patients, rather than opening up a more inclusive perspective on the person's needs, because opening up this dialogue may result in a disappointed patient who is more aware about what they are missing?*

Interestingly, the distribution of QPR scores showed similar characteristics to the sample of 942 consumers of specialist mental health services receiving a similar intervention (15). Experiences of personal recovery may therefore be similarly reported by people accessing different levels of care. This finding is an important contribution to the growing evidence base on self-reported measures of participant experience and potentially provides evidence for using the QPR survey within primary care, which is where the majority of mental health conditions are treated.

There are several important limitations that need to be taken into account in interpreting the results as they contain threats to validity (32, 36). Firstly, there was no concurrent control group, and other factors occurring over time may have contributed to the outcomes (32). Secondly, the cross-sectional pre and post data used different patient groups, also the case in the mentioned secondary care study (15). Although the two samples were reasonably similar on most demographic characteristics, it is possible that the second sample happened to be a group with less severe mental health issues (as indexed by fewer people being on medications) and the presence of this bias is why their results were better. Alternatively, it is also possible that ROP led to reduced need for medications. Across the PULSAR program we adopted this strategy with concerns that in the time frame of the intervention patients benefitting from ROP might leave the service setting for a lower level of care (15) or as here, for self-care or alternative support settings. Longitudinal designs could be preferable but even more challenging to implement.

Despite these limitations, there is useful learning from this trial. The development of evidence-based interventions is a complex undertaking involving many stages (37). The evaluation of ROP in primary care is in its infancy as this is, to our knowledge, the first trial of a ROP staff development intervention delivered in primary care settings. Although we were not successful in implementing the initial project plan as set out in the protocol paper, we adapted the design to enable the exploratory collection of observational data, the findings for which were positive on a number of variables. However, given the context of the altered design with its inherent risk of bias, further research is needed. Although there is some encouragement from these findings, at least as important as the findings at this initial stage of the research is what we learned in our efforts to implement this trial, which can inform future studies.

Barriers are known to reduce participation in research for Australian GPs (38) and the most common obstacle cited is that of limited time (39–41) including attrition attributed to competing demands of time-poor GPs' (39). In implementing the trial, this barrier was well-understood from the start. This was a well-resourced project and we used many flexible strategies to support the engagement and recruitment efforts of GPs as described in the protocol paper. Nevertheless, the requirement for GPs to recruit patients for the trial, despite significant financial and practical support from project staff (19), was for the most part not something they were equipped to do effectively. Compounding this problem was the cross-sectional design. Although the design was highly effective in our specialist care trial in meeting our recruitment targets (15), it was less workable in primary care as it increased the burden on GPs further since they needed to recruit new participants at multiple time points.

Notwithstanding the challenges of GPs having the time to engage in research, prior work indicates that both they and their patients are motivated to participate in research for the knowledge gained, improved patient outcomes and altruistic benefit to others (16, 40, 42, 43), as also shown in this study although not to the originally expected level. What would have been most helpful to the success of this project would be to have an established network of research-oriented GPs connected with a health and mental health service and who are equipped to conduct research. This project essentially was trying to create these networks of research-engaged practices while simultaneously running the project—with initial GP recruitment largely involving cold-calling over 325 practices (19). We note though that such service settings might represent an efficacy testing context rather than effectiveness since the practices would not be representative. But this could be an important step in the translational research process. The situation in primary care stands in stark contrast to the specialist care study which had the same overall project design. The latter study was highly successful in its implementation of the project plan and this largely rested on the already established network of research relationships between each of the participating organizations. This context flags the importance of infrastructure development in recruitment, engagement and establishing shared priorities in primary care research. The current investment in the academic health sciences centers should ensure that primary health care is sufficiently well-engaged to result in these kinds of network developments.

ROP is gaining increasing prominence in mental health service delivery and the outcomes of such an approach within the primary care sector for the first time is investigated in this project. The findings suggest this trend is justified given the intervention reported here showed potential benefit to patients when ROP training was extended to GPs throughout the community. Nevertheless, challenges in implementing the project means that the findings are subject to bias and flag the importance establishing research infrastructure in primary care. This study outlines (and suggests improvements for) a feasible method of implementing and assessing a pragmatic intervention to encourage ROP for mental health in GP settings.

## Summary

### Strengths and Limitations of This Study

- Recovery-oriented practice, well-established in specialist mental health service and with developing evidence of effectiveness of training interventions, lacks a body of research work in the critically important primary mental health care setting; this study adds positive exploratory findings for patient-rated personal recovery following a primary care setting recovery-oriented practice training intervention.
- Implementation challenges included recruitment, sustainability of engagement and protocol adherence. An initially ambitious stepped-wedge cluster RCT, successfully implemented in the secondary care arm of the project, was necessarily revised to a pretest–posttest design in the primary care arm.
- Reflections on these implementation challenges will inform initiatives to establish and maintain collaborative primary mental health care research infrastructures.

## Data Availability Statement

The datasets presented in this article are not readily available because in compliance with the requirements of the Monash Health Research Ethics Committee, the data supporting our findings in the article will not be shared because we did not obtain participant consent to do so. Requests to access the datasets should be directed to Graham Nicholas Meadows, graham.meadows@monash.edu.

## Ethics Statement

The studies involving human participants were reviewed and approved by Monash Health Research Ethics Committee. The patients/participants provided their written informed consent to participate in this study.

## Author Contributions

GM was the principal investigator and, together with JE, led development of key elements of the design and analysis approach and interpretation of the findings. MS developed the original REFOCUS intervention and advised on its adaptation. LB chaired the research module task group and provided oversight to development and implementation of all elements of the design. FS provided overall coordination for survey distribution and staff training and was centrally involved in the day-to-day operations of trial implementation. JE conducted the data analyses. EW-E made specialist contributions to certain elements of study design. VE, GR, DM, LB, GM, and EW-E developed the training intervention and associated resources. JE, FS, LB, and GM comprised the core drafting group. All authors commented on the final draft.

## Conflict of Interest

The authors declare that the research was conducted in the absence of any commercial or financial relationships that could be construed as a potential conflict of interest.
