# Supplementary material for: REFOCUS-PULSAR Recovery-Oriented Practice Training in Adult Primary Mental Health Care: Exploratory Findings Including From a Pretest–Posttest Evaluation
Source: Front Psychiatry. 2021 Mar 11;12:625408. doi: 10.3389/fpsyt.2021.625408 (PMC8006334; doi:10.3389/fpsyt.2021.625408)
Supplement: Supplementary file 1 [file Image_1.pdf]

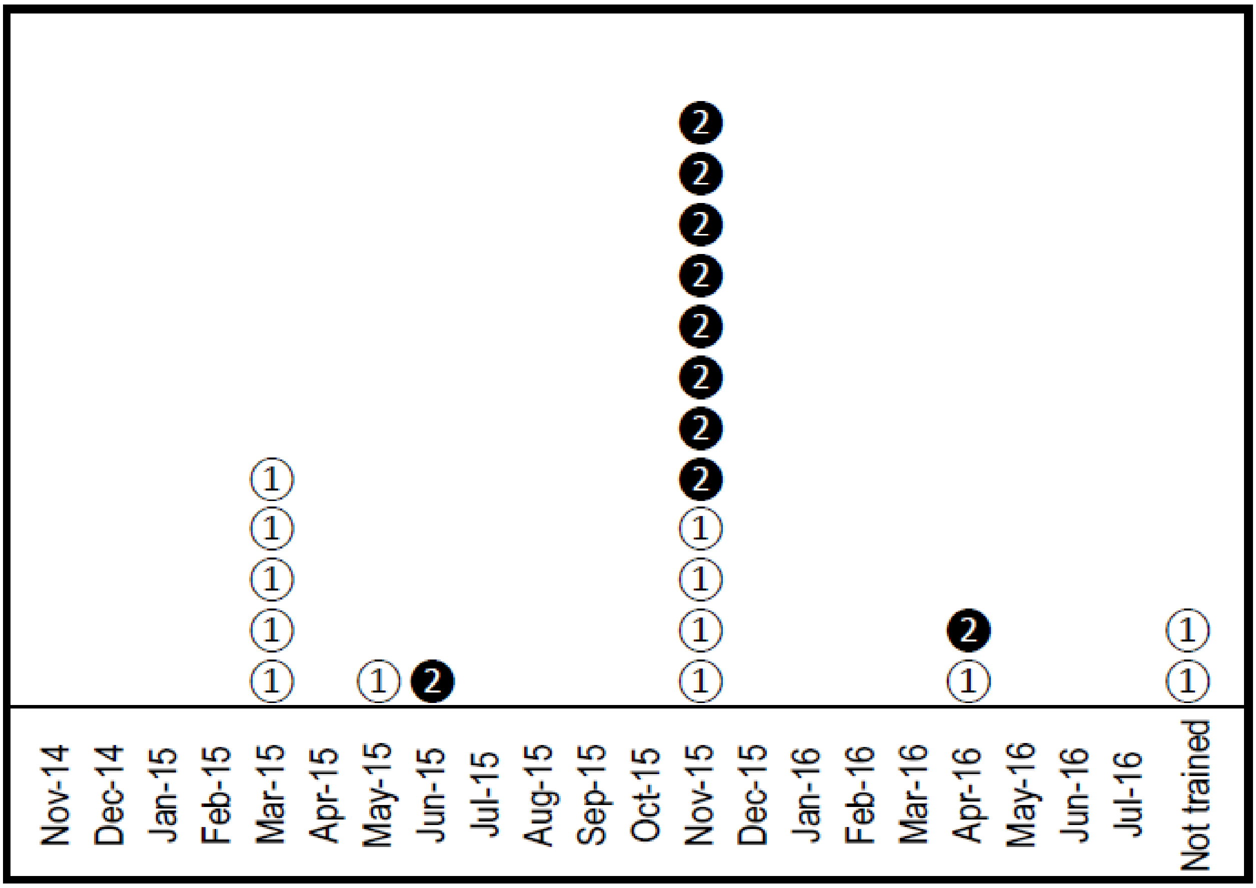

Supplementary file 1. Training dates of the 23 participating General Practitioners (GP). Each circle is a GP. White circles are GPs randomly allocated to step 1 (early) training period. Black circles are GPs allocated to step 2 (late) period. GPs in step 1 were planned to undergo training first, and then approximately 9 months later, the step 2 GPs trained. However, as shown in the figure, two training periods did not eventuate, and often GPs in either group trained together. This clearly shows the original stepped-wedge cRCT intervention design was not achieved.
