# Supplementary material for: REFOCUS-PULSAR Recovery-Oriented Practice Training in Adult Primary Mental Health Care: Exploratory Findings Including From a Pretest–Posttest Evaluation
Source: Front Psychiatry. 2021 Mar 11;12:625408. doi: 10.3389/fpsyt.2021.625408 (PMC8006334; doi:10.3389/fpsyt.2021.625408)
Supplement: Supplementary file 2 [file Image_2.pdf]

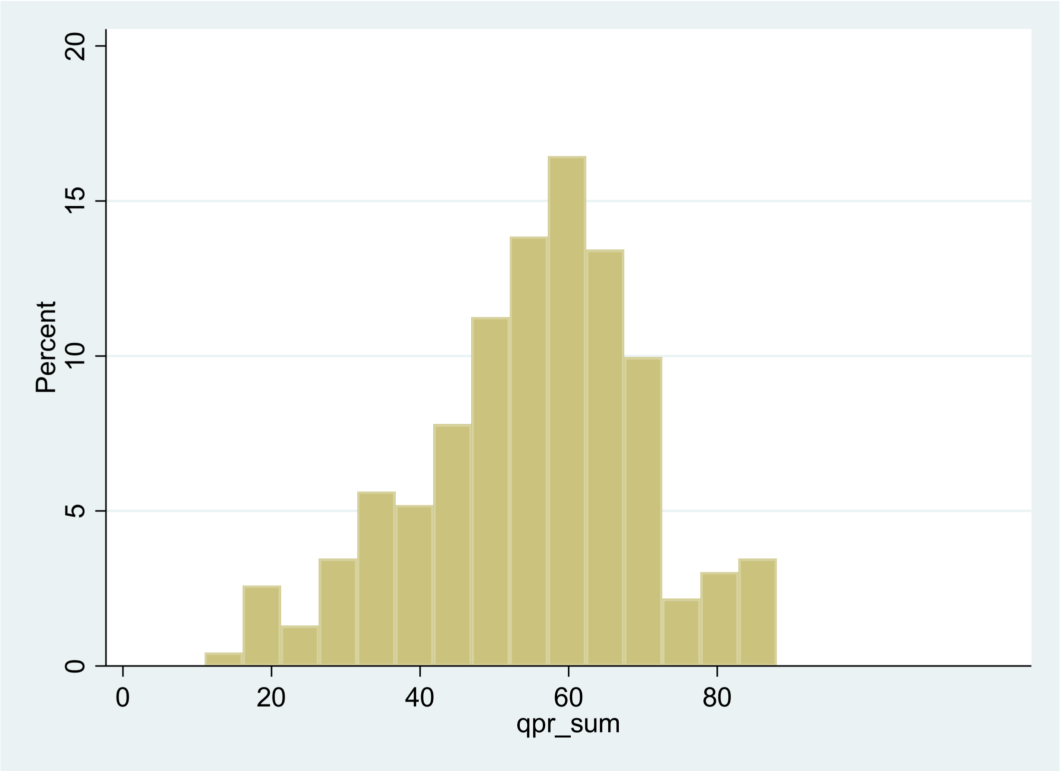

Figure A. Outcome distribution of QPR scores from all 235 patients in this primary care trial. QPR mean of 54.7, standard deviation of 15.2 (possible score ranges between 0 and 88).

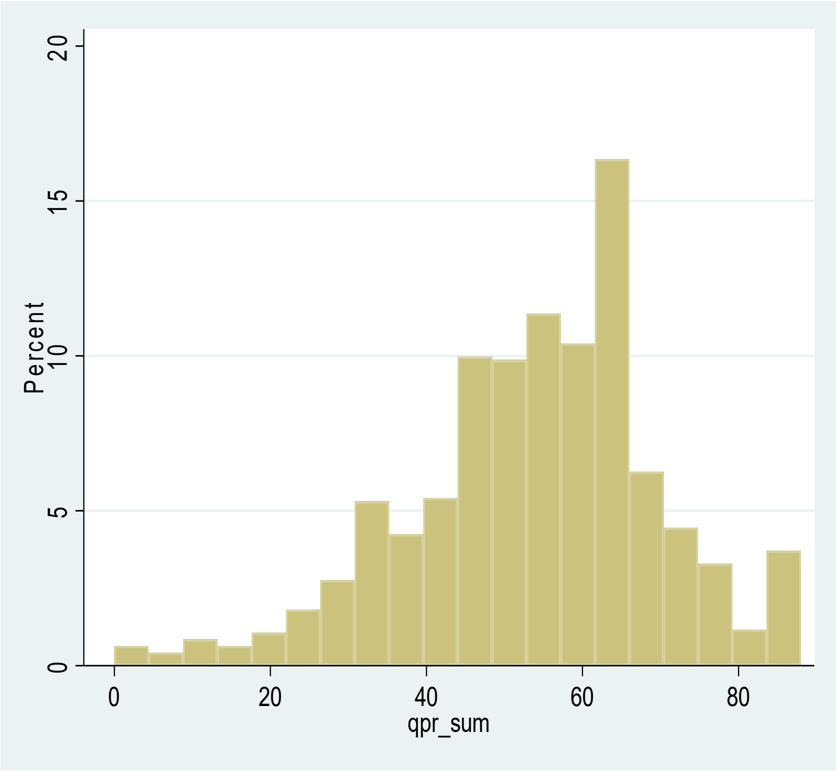

A two-sample Kolmogorov-Smirnov test for equality of distribution showed that these QPR datasets (A and B) appear to have the same distribution function (D-statistic= 0.047, *p*<0.80).

Figure B. Outcome distribution of QPR scores from all 942 patients in secondary care from the trial by Meadows et al., (2019). QPR mean of 54.0, standard deviation of 16.2 (possible score ranges between 0 and 88).
