# Supplementary material for: REFOCUS-PULSAR Recovery-Oriented Practice Training in Adult Primary Mental Health Care: Exploratory Findings Including From a Pretest–Posttest Evaluation
Source: Front Psychiatry. 2021 Mar 11;12:625408. doi: 10.3389/fpsyt.2021.625408 (PMC8006334; doi:10.3389/fpsyt.2021.625408)
Supplement: Supplementary file 3 [file Image_3.pdf]

Supplementary file 3:

For the primary outcome, propensity scores were estimated by the conditional treatment probability using maximum likelihood (probit regression), with independent factors of GP clinic (cluster), gender, age, country of birth (Australia: Yes/No). Propensity score and Mahalanobis matching drawn with replacement were was applied using the stata code PSmatch2. Four matching strategies were investigated:

- Nearest neighbour 1-to-1
- Nearest neighbour 1-to-2
- Mahalanobis 1-to-1
- Mahalanobis 1-to-2

These matching strategies showed slightly larger intervention effects that were significant at p<0.05; however, 10-13% of the available data was excluded from these analyses, (i.e. in the available QPR primary outcome data from 235 subjects, the four matched analyses had sample sizes of: 206, 206, 210 and 208 respectively). Incomplete matching particularly when done with relatively small data sets such as in this paper, has been reported to contribute towards biased treatment estimates (Caliendo and Kopeinig 2008; Yao et al., 2017). Therefore, the pre- and post- design was chosen as the main analysis because it used all the available data.

Table Supplementary. Pre and post (PP) intervention outcome differences determined using propensity score matching analyses.* Mean difference significant at p<0.05

		Pre-Ix Mean	Post-Ix Mean	Mean difference	standard error	t-statistic
QPR	unmatched	52.8	57.3	-4.4*	1.99	-2.23
	Nearest neighbour 1-to-1	52.8	59.3	-6.5*	2.66	-2.44
	Nearest neighbour 1-to-2	52.8	58.7	-5.9*	2.29	-2.57
	Mahalanobis 1-to-1	52.8	58.7	-6.0*	2.40	-2.48
	Mahalanobis 1-to-2	52.8	58.5	-5.7*	2.19	-2.60

Yao, X. I., Wang, X., Speicher, P. J., Hwang, E. S., Cheng, P., Harpole, D. H., Berry, M. F., Schrag, D., & Pang, H. H. (2017). Reporting and Guidelines in Propensity Score Analysis: A Systematic Review of Cancer and Cancer Surgical Studies. Journal of the National Cancer Institute, 109(8), djw323. https://doi.org/10.1093/jnci/djw323

Caliendo M, Kopeinig S.. Some practical guidance for the implementation of propensity score matching. J Econ Surv. 2008;221:31–72.
